# Case Report: Use of Subcutaneous Midazolam During an Episode of Catatonia

**DOI:** 10.3389/fpsyt.2021.666646

**Published:** 2021-04-14

**Authors:** Valentin Raymond, Etienne Véry, Adeline Jullien, Fréderic Eyvrard, Loic Anguill, Antoine Yrondi

**Affiliations:** ^1^Department of Psychiatry, Psychotherapy, and Art therapy, University Hospital Toulouse (CHU Toulouse), Toulouse, France; ^2^ToNIC Toulouse NeuroImaging Center, Toulouse University, INSERM, UPS, Toulouse, France; ^3^Department of Pharmacy, University Hospital Toulouse (CHU Toulouse), Paule de Viguier Hospital, Toulouse, France; ^4^Department of Psychiatry and Medical Psychology, University Hospital Toulouse (CHU Toulouse), Toulouse, France; ^5^Department of Psychiatry and Medical Psychology, University Hospital Toulouse (CHU Toulouse), Expert Centre for Treatment-Resistant Depression FondaMental, ToNIC Toulouse NeuroImaging Centre, Toulouse University, INSERM, UPS, Toulouse, France

**Keywords:** catatonia, benzodiazepine, subcutaenous administration, negativism, withdrawal

## Abstract

Midazolam is a benzodiazepine (BZD) mainly used in anesthetic induction due to its pharmacokinetic features. Its place in the therapeutic management of catatonia remains to be determined. Here we present the case of a 65-year-old man who presented with a first episode of catatonia with opposition to any form of oral treatment, where a single dose of 1 mg of subcutaneous (SC) Midazolam permitted clinical improvement allowing oral treatment to be given. The patient's history notably included a renal transplant linked to Polycystic Kidney Disease (PKD) and no history of psychiatric illness nor of any use of psychotropic drugs. As the patient refused to drink or eat and ceased answering basic questions, a psychiatric assessment was required. A diagnosis of Catatonic disorder due to a general medical condition [DSM 5–293.89/ ICD10 [F06.1]] was made. A Bush-Francis Catatonia Rating Scale (BFCRS) analysis returned a score of 15 out of 62, with stupor, mutism, negativism, staring, withdrawal, rigidity, and stereotypy. As the negativism prevented the patient from taking any form of oral treatment, after a brief discussion with the unit's physician, it was decided to administer 1 mg of SC Midazolam. One hour later, the patient was more responsive and compliant, and agreed to drink, eat, and take medication. Thus, the catatonic signs of mutism, negativism, staring, and withdrawal were resolved, but waxy flexibility and catalepsy appeared, leading to a new BFCRS score of 10 out of 62. Oral treatment with 2.5 mg Lorazepam, 4 times a day, was then initiated. Midazolam could be a safer choice compared with the other options available, such as other SC BZD, considering the complex safety profile of this patient with renal insufficiency. This situation represents the first report of using SC Midazolam as an injectable treatment for catatonia. More studies are needed to assess the clinical pertinence of SC Midazolam in the treatment of catatonia.

## Case Report

### Background

The incidence of catatonia in patients aged over 65 in medical departments associated with seeking the advice of a psychiatry liaison service can be as high as 8.9% (1). It is now well-known that the first-line treatment for catatonia is oral benzodiazepine (BZD), with a preference for Lorazepam in view of its pharmacokinetic properties (2, 3).

However, the implementation of these recommendations may be hampered by the catatonic symptoms themselves. Negativism and withdrawal are found in almost half of catatonia cases (4). Such symptoms often prevent the patient from accepting any form of oral treatment, and encourage clinicians to find alternative routes for BZD administration. Only a few case reports have previously described that intravenous (IV) administration of Midazolam could help diagnose catatonia (5) or alleviate its symptoms (6–9). But as far as we know, the successful use of subcutaneous (SC) Midazolam as a treatment for catatonia has never been reported. We present the case of a 65-year-old man who presented with a first episode of catatonia with opposition to any form of oral treatment, where the use of a single dose of SC Midazolam provided sufficient improvement in catatonic symptoms to allow oral treatment to be given.

### Case Report

The case presented below concerns a 65-year-old man, whose medical history includes a renal transplant linked to Polycystic Kidney Disease (PKD), with hepatitis C, subsequent compensated cirrhosis with a Child-Pugh score of A6, and insulin-dependent diabetes. The patient had no history of psychiatric illness, *nor substance abuse or* any use of psychotropic drugs. He weighed 67 kilograms (kg), and was 66 inches tall, with a body mass index of 24 kg/square meters.

He presented in the Toulouse-Rangueil University Hospital Centre's emergency department with confusion. A blood sample confirmed hyponatremia of 119 millimoles per liter (mmol/L) and the patient was subsequently transferred to a specialized transplant unit [UTO].

This hyponatremia was considered likely to be due to the recently introduced diuretic treatment with Amiloride.

A few days later, despite an improvement in the ion channel disorders following a diminution of water intake, and a period of good contact with medical staff or family, the patient suddenly refused any form of care, including taking his immunosuppressive treatment. He also refused to drink or eat, and stopped answering basic questions.

His clinical state consequently worsened with aggravation of his chronic renal failure, with an increase in creatinine blood level from 270 to 300 mcmol/L, and a decrease in Glomerular Filtration Rate from 21 to 18 mL/min.

A brain scan was requested, the results of which showed no anomaly.

In these circumstances, psychiatric advice was requested from the Liaison Psychiatric Unit as a case of diagnosis emergency.

A diagnosis of Catatonic disorder due to a general medical condition [(DSM 5–293.89/ICD10 [F06.1]] (10, 11) was made. A Bush-Francis Catatonia Rating Scale (BFCRS) (12) investigation returned a score of 15 out of 62, with stupor, mutism, negativism, staring, withdrawal, rigidity, and stereotypy.

As the negativism prevented the patient from taking any form of oral treatment, after a brief discussion with the unit's physician, it was decided to administer 1 mg of SC Midazolam.

One hour later, the patient was more responsive and compliant, and agreed to drink, eat, and take medication. Thus, the catatonic signs of mutism, negativism, staring, and withdrawal were resolved, but waxy flexibility and catalepsy appeared, leading to a new BFCRS score of 10 out of 62.

Following this, oral treatment with 2.5 mg Lorazepam, 4 times a day, was initiated and injectable Midazolam was not repeated. The patient quickly improved, continued to drink and eat without trouble, and complete remission of the catatonic syndrome was achieved in 6 days. This *oral* treatment was continued for 5 days, then quickly reduced to 5 mg a day for 4 days and finally stopped.

Medical tests seeking the possible etiology of this catatonia, such as a cerebral MRI scan or an electroencephalogram, came back without any anomaly that could explain this symptomatology.

The patient was then discharged from the medical unit and returned home.

### Discussion

To our knowledge, this situation represents the first report of using SC Midazolam as an injectable treatment for catatonia. SC administration is not the only alternative route for BZD administration when catatonic symptoms, such as negativism and withdrawal, are present and preventing the patient from accepting any form of oral treatment. Among these alternatives, injectable BZD can be an option during the period of patient withdrawal. Indeed, when patients are opposed to oral treatment due to catatonic signs, injectable treatment (intravenous, intramuscular, or subcutaneous) can quickly improve symptoms and allow either subsequent oral treatment or full recovery from the catatonic syndrome.

Intramuscular (IM) or intravenous (IV) Lorazepam is the most widely used treatment and recommended as first-line treatment in situations of catatonia with opposition to oral treatment (2, 3).

However, in several countries IM Lorazepam is unavailable or, as in France, difficult to obtain, particularly in emergency situations.

Additionally, IM or IV injections can present problems in terms of tolerance or practicality.

Apart from being painful, intramuscular injections present several other disadvantages such as a risk of bleeding for patients with haemostasis disorders, nerve damage, or haematoma formation.

Intravascular injections share the same concerns considering haemostasis inconveniences, and also require venous access, which can be more harmful for patients by exposing them, for example, to a higher risk of infection or to more stress.

Moreover, patients with conditions such as catatonia could present with a state of agitation, which may lead to venous access failing, leaving patients at risk of traumatic injuries and to non-delivery of the treatment.

In an emergency situation involving agitation, for example, IV injection might not be the best option, as the insertion of a catheter for treatment administration may be impossible.

Nevertheless, SC injections present several advantages over other types of injection. They are easier to administer with no skilled personnel needed, quicker to use as they don't require venous access, and have a better tolerance profile. Indeed, such injections are generally less painful or distressing for the patient, the risk of infection is lower and generally localized instead of generalized, the needle-stick injury risk for nursing staff is lower, reducing blood exposure accident risk, and contraindications in case of haemostasis disorders are only relative (13, 14).

A few BZDs are available in SC form, such as Diazepam, Clorazepate, or Clonazepam. Among them, the best tolerance profile is offered by SC Midazolam, the use of which as an alternative injectable BZD with catatonic patients may be of interest and has not yet been studied.

This molecule is clinically relevant due to its pharmacokinetic characteristics, such as a short elimination half-life, unlike other SC BZDs, a rapid attainment of the maximum blood concentration, and then a rapid onset of action regardless of the form in which it is presented. These characteristics allow a quick diagnostic confirmation with symptomatology improvement. Moreover, this improvement can lead to patient acceptance of an oral BZD, which then simplifies administration.

In the case presented above, Midazolam could also be a safer choice compared with the other available SC BZDs considering the complex safety profile of this patient with renal insufficiency (15).

We can wonder whether the dose used in this report can be estimated as low, with only 1 mg of Midazolam administrated to the patient.

As indicated in the Summary of Product Characteristics (SPC), in adults over the age of 60 or in a poor general condition, initial dose should be reduced to 0.5 or 1 mg. Moreover, here, we had to choose wisely how to use this medication, the patient presenting with an acute aggravation of his chronic renal failure, subsequent to his already poor welfare initially.

These warning signs led us to err on the side of caution in choosing the dosage of BZD, and encouraged us in the choice of a quickly metabolized and eliminated one, such as Midazolam.

More studies are needed to assess clinical pertinence, focusing on dose chosen and the safety of SC Midazolam in the treatment of catatonia.

Moreover, more needs to be done in the struggle to enhance psychiatric knowledge concerning catatonia and its treatment, especially injectable treatment, as situations where oral treatment is not possible can be frequently encountered.

## Data Availability Statement

The raw data supporting the conclusions of this article will be made available by the authors, without undue reservation.

## Ethics Statement

The studies involving human participants were reviewed and approved by MR004 CHU TOULOUSE. Written informed consent for participation was not required for this study in accordance with the national legislation and the institutional requirements.

## Author Contributions

VR, EV, LA, and AY were focusing on catatonia. AJ and FE were focusing on treatment. All authors participated to the drafting of the final manuscript.

## Conflict of Interest

The authors declare that the research was conducted in the absence of any commercial or financial relationships that could be construed as a potential conflict of interest.
